# Air pollution and suicide mortality in the UK: implications for environmental and mental health policy

**DOI:** 10.1007/s11356-026-38089-w

**Published:** 2026-08-01

**Authors:** Sara Daoud, Alaa Al-Aswadi, Humayd Dada, Rima J. Isaifan

**Affiliations:** School of Foreign Service, Georgetown University, P.O. Box 23689, Doha, Qatar

**Keywords:** PM₂.₅, Air pollution, Suicide, Mental health, Policy, UK

## Abstract

Air pollution is a well-established risk factor for physical illness and premature mortality, yet its relationship with suicide remains less well understood, particularly at the national level and over longer periods of exposure. Although previous studies have reported positive associations between particulate matter and suicide, evidence remains heterogeneous, and national longitudinal analyses are limited. This study examines the association between fine particulate matter (PM₂.₅) concentrations and suicide mortality across local authorities in the UK between 2015 and 2023. Suicide data obtained from the UK Office for National Statistics were combined with modelled PM₂.₅ estimates from the Department for Environment, Food and Rural Affairs, resulting in a balanced panel of more than 2200 local authority-year observations. Fixed-effects panel regression models were employed to account for unobserved time-invariant local characteristics and common year-specific shocks, while nonlinear specifications were estimated to examine potential threshold effects. The descriptive analysis revealed a negative cross-sectional association between PM₂.₅ concentrations and suicide rates. However, after controlling for local authority and year fixed effects, PM₂.₅ was positively and significantly associated with suicide mortality (*β* = 0.221, *p* = 0.007). The nonlinear model further identified a statistically significant U-shaped relationship, with an estimated turning point at approximately 7.8 µg/m^3^, suggesting that higher PM₂.₅ concentrations are associated with increased suicide mortality beyond this threshold. Although the nonlinear specification modestly improved model fit, the findings should be interpreted cautiously given the observational design and the absence of certain time-varying socioeconomic controls. Overall, the results suggest that the relationship between PM₂.₅ and suicide mortality is conditional rather than uniform, varying across pollution exposure levels and geographic contexts. While the findings provide national-scale evidence on the association between long-term PM₂.₅ exposure and suicide mortality in the UK, they should be interpreted as exploratory associations rather than evidence of causality. Future research incorporating additional socioeconomic variables, longer time series, and higher temporal resolution data is needed to further evaluate these relationships.

## Introduction

Air pollution is a well-established factor contributing to both illness and premature death, with particularly strong links to heart and lung diseases (Al-Thani and Isaifan 2025; Isaifan 2023; Isaifan and Al-Thani 2024). However, its potential role in influencing suicide has received comparatively little academic attention (Ng et al. 2016). In the UK, suicide rates have increased in recent years, even while national levels of fine particulate matter (PM₂.₅), airborne particles smaller than 2.5 µm, have steadily declined (ONS 2025). This contrast raises an important question: Do regions that continue to experience higher pollution levels also exhibit elevated suicide rates compared to cleaner areas?

Recent evidence has strengthened the hypothesized association between air pollution and suicide. A comprehensive systematic review and meta-analysis by Heo et al. (2021) concluded that exposure to PM₂.₅, PM₁₀, and NO₂ were associated with an increased risk of suicide, whereas no significant associations were observed for O₃, SO₂, or CO (Heo et al. 2021) . The review also identified important research gaps, including the need for studies examining different geographical contexts, long-term exposure patterns, and the interaction between environmental and contextual factors. These findings highlight that, despite growing evidence, important uncertainties remain regarding the relationship between air pollution and suicide, particularly at broader spatial scales and across diverse populations. Much of the existing literature has focused on short-term exposure events, single metropolitan areas, or cross-sectional comparisons, leaving relatively limited evidence on whether within-area changes in long-term PM₂.₅ exposure are associated with suicide mortality at the national level. This study addresses this gap by analyzing local authority panel data across the UK using fixed-effects regression models that account for unobserved time-invariant heterogeneity. In addition, the study examines whether the association is nonlinear across different pollution levels. Rather than testing a causal mechanism, the study provides national-scale evidence on how temporal changes in PM₂.₅ exposure are associated with suicide mortality after accounting for persistent regional characteristics and common temporal shocks.

There are biological mechanisms that make such a connection plausible. Due to their small size, PM₂.₅ particles can reach the lungs, pass into the blood, and eventually make their way across the brain’s protective barrier (Flood-Garibay et al. 2023; Hu et al. 2024). Once inside the nervous system, they may provoke inflammation, oxidative stress, and disturbances in neurotransmitter function, processes that have been linked to mood disorders and suicidal (Block and Calderón-Garcidueñas 2009; Fonken et al. 2011).

Epidemiological studies provide empirical support for this link. For example, Kim et al. (2010) found that daily rises in particulate matter concentrations in Seoul were linked to higher suicide cases (Kim et al. 2010), and Cho et al. (2014) observed similar patterns across multiple South Korean cities (Cho et al. 2014). In the UK, Sun et al. (2020) showed that London boroughs with higher annual PM₂.₅ levels also experienced higher suicide rates, even when socioeconomic deprivation was considered (Sun et al. 2020). Collectively, these findings suggest that air pollution is associated with both short-term increases in suicide risk and longer-term regional variation in suicide mortality.

The UK is a compelling setting to revisit this question. While national policy has driven down average PM₂.₅ concentrations, regional disparities remain marked: central London boroughs, for example, continue to record annual averages well above those in rural areas (Sun et al. 2020). This uneven exposure creates an opportunity to test whether suicide rates are systematically higher in areas that remain more polluted, regardless of the broader downward trend in air quality. Sun et al.’s (2020) London-based study is a key point of departure. Their analysis underscored that spatial variation in exposure may play a large role as short-term pollution spikes in shaping mental health outcomes (Sun et al. 2020).

Building on prior research, this study extends the analysis to a national scale. It hypothesizes that persistently elevated levels of fine particulate matter (PM₂.₅) are associated with higher suicide mortality. Specifically, it posits that local authorities with greater exposure to air pollution will exhibit higher suicide rates, even after accounting for unobserved structural characteristics and common temporal shocks. To test this, the study compares the ten highest- and low-exposure regions and examines within-area changes over time. This approach enables an assessment of whether spatial and temporal variation in air quality continues to shape suicide outcomes across the UK, despite overall improvements in national air pollution levels.

## Methods

This study uses deaths by suicide data provided by the UK ONS, combined with modeled PM₂.₅ exposure data produced by DEFRA (Department for Environment, Food, and Rural Affairs 2026; ONS 2025). The dependent variable is the suicide rate per 100,000 individuals, age-standardized, and reported using 3-year rolling averages at the local authority level. These rolling averages help reduce short-term fluctuations in smaller populations while preserving meaningful regional and temporal variation in suicide outcomes. For this research, the middle year of each 3-year average was used to align the data consistently across time. The key independent variable is the annual mean PM₂.₅ concentration (µg/m^3^), which is obtained from DEFRA’s Pollution Climate Mapping (PCM) model. This model incorporates national emissions inventories, atmospheric dispersion dynamics, and calibration with monitoring station data to generate consistent estimates at the local authority scale.

The ONS suicide data and DEFRA air quality estimates were harmonized into a single balanced panel dataset covering the period 2015–2023. Local authority codes were standardized across both sources to enable merging, and the resulting dataset contained over 2200 authority-year observations. Each record thus captures the suicide rate and PM₂.₅ concentration for a given local authority in a given year. Data cleaning involved reshaping the ONS suicide tables so that rolling averages matched the appropriate calendar years, checking for consistency in local authority boundaries, and systematically assessing missing values. The final dataset included 252 local authorities, covering both urban boroughs with persistently high exposure (e.g., Westminster, Camden) and rural areas with some of the lowest concentrations in the country.

The empirical design aimed to evaluate whether persistently elevated PM₂.₅ concentrations are linked to higher suicide mortality, even as national air quality has improved. To begin, descriptive statistics and simple correlations were used to provide an initial picture of the association between pollution and suicide, including contrasts between the most and least polluted local authorities. Building on this preliminary analysis, the core statistical approach employed was fixed-effects panel regression (Stata command: xtreg, fe). This method controls for unobserved, time-invariant characteristics specific to each local authority. By doing so, it accounts for structural factors such as geographic location, long-term deprivation, and disparities in health service provision. Year fixed effects were also incorporated to capture nationwide shocks and temporal patterns.

Although year fixed effects account for nationwide temporal shocks affecting all local authorities simultaneously, the analysis does not include local authority–specific time-varying socioeconomic variables such as unemployment, housing stress, mental health service availability, or substance use. Consistent annual data for these indicators was not available across the full study period. Consequently, the estimated coefficients should be interpreted as within-area associations rather than causal effects.

A range of model specifications was applied. The baseline fixed-effects regression estimated the linear relationship between PM₂.₅ exposure and suicide rates across local authorities. To account for possible nonlinearities, an alternative specification added a squared term for PM₂.₅, allowing the effect to vary across different exposure levels. This made it possible to test whether suicide risk followed a threshold effect or a U-shaped curve, as proposed in earlier environmental health research.

The analysis focuses on contemporaneous annual associations between PM₂.₅ concentrations and suicide mortality. Although lagged effects are biologically plausible, the relatively short annual panel limited the feasibility of estimating reliable lagged or distributed-lag models.

In addition, first-difference models were employed, regressing year-to-year changes in suicide rates against changes in PM₂.₅ concentrations. This more conservative strategy relied exclusively on temporal variation within authorities, helping to minimize bias from time-invariant unobserved characteristics.

All estimations incorporated robust clustered standard errors at the local authority scale to address possible heteroskedasticity and autocorrelation within clusters. Model performance was evaluated using within *R*^*2*^ values and intra-class correlation coefficients, which reveal the extent to which variation is explained by time-varying factors versus persistent regional differences. Considerations of statistical significance, effect size, and substantive interpretability guided the evaluation of findings.

Finally, the methodological choices reflect the policy relevance of the research. Although national average PM₂.₅ exposure has declined, regional disparities remain entrenched. Urban boroughs such as Newham, Tower Hamlets, and Camden continue to experience higher concentrations than rural authorities in Wales or the Scottish Highlands. By focusing on these within-country inequalities, the study evaluates whether persistently exposed regions also face a disproportionate burden of suicide. This approach situates the analysis within an environmental justice framework. If higher pollution levels are linked to worse mental health outcomes, then vulnerable communities face compounded risks that require targeted interventions in both air quality management and suicide prevention policy.

## Results

**Fig. 1:**
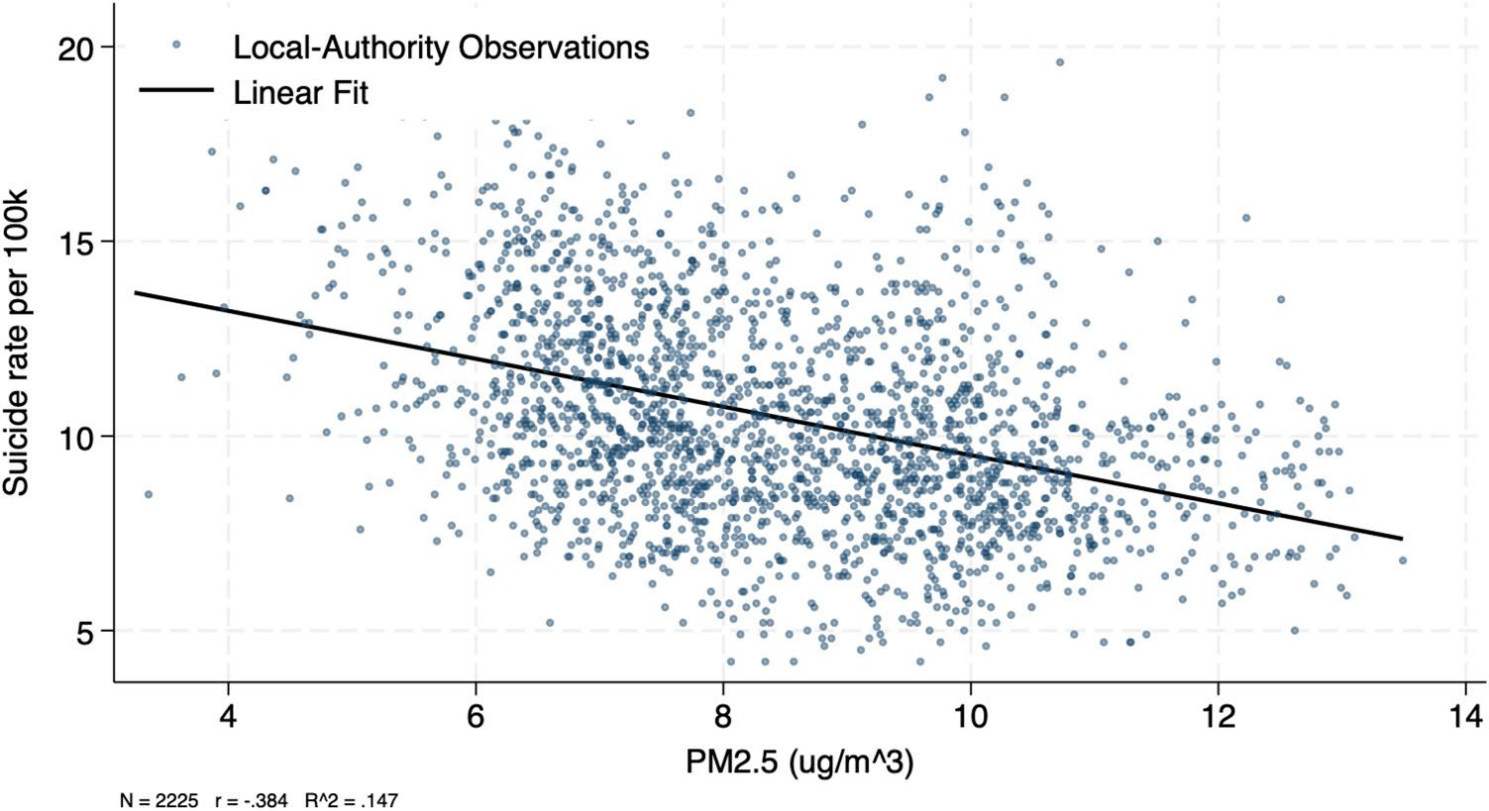
Scatter plot of the PM_2.5_ concentration vs suicide rate during 2015–2023

Figure 1 presents the unconditional relationship between PM₂.₅ concentrations and suicide rates across UK local authorities. The raw data shows a negative bivariate association, illustrating the importance of accounting for unobserved regional heterogeneity in the subsequent fixed-effects analyses. The estimated correlation coefficient (*r* = −0.384) suggests a moderately strong negative association. The corresponding coefficient of determination (*R*^*2*^ = 0.147) indicates that approximately 15% of the variation in suicide rates across authorities and over time can be explained by PM₂.₅ concentrations in this bivariate framework.

Table 1 summarizes the distribution of PM₂.₅ concentrations and suicide rates across the study period. Although national air quality improved over time, substantial regional variation remained, providing sufficient within-country variability for the panel analyses. Suicide mortality also exhibited considerable variation across local authorities, supporting the use of fixed-effects models to distinguish within-area temporal associations from persistent regional differences.

**Table 1:** Descriptive statistics for PM₂.₅ concentrations and suicide rates (2015–2023)

Variable	Observations	Mean	Standard deviation	Min	Max
PM₂.₅ (µg/m^3^)	2237	8.47	1.77	3.24	13.61
Suicide rate	2225	10.46	2.83	4.20	20.60

As per Table 1, the mean PM₂.₅ concentration across local authorities was 8.47 µg/m^3^, with a standard deviation of 1.77. On average, this level is close to the World Health Organization’s earlier guideline of 10 µg/m^3^, but exceeds the stricter 2021 recommendation of 5 µg/m^3^ (Pai et al. 2022). The lowest recorded concentration was 3.24 µg/m^3^, typical of the cleanest rural locations, while the highest value reached 13.61 µg/m^3^, a pattern characteristic of densely populated or traffic-heavy boroughs in central London. These figures demonstrate that, although national air quality has generally improved, substantial inequalities remain. Certain regions continue to experience persistently higher levels of particulate exposure, highlighting the uneven distribution of environmental health risks.

The mean suicide rate over the study period was 10.46 cases per 100,000 population, with a standard deviation of 2.83, indicating moderate variability across local authorities and over time. This average closely aligns with national figures reported by the Office for National Statistics, suggesting that the dataset is representative of broader population trends. The observed range is substantial, with rates varying from a minimum of 4.2 to a maximum of 20.6 per 100,000 population. While the lower bound reflects areas with relatively low suicide burden, the upper bound indicates local authorities where suicide mortality exceeds twice the national average. This dispersion highlights pronounced regional inequalities in mental health outcomes, likely driven by underlying differences in socioeconomic conditions, access to healthcare services, and other social determinants of health.

Table 2 reports the results of the fixed-effects regression analysis examining the relationship between PM₂.₅ concentrations and suicide mortality across 252 UK local authorities over the period 2015–2023. The fixed-effects specification controls for unobserved, time-invariant characteristics at the local authority level, such as geographic features, structural deprivation, and baseline health system capacity, thereby isolating the effect of within-area variation over time. Robust standard errors clustered at the local authority level were employed to account for serial correlation and heteroskedasticity arising from repeated observations.

**Table 2:** Fixed-effects regression of suicide rates on PM₂.₅ (2015–2023)

	Coefficient	Standard Error	*t*	*p*-value	95% CI
PM₂.₅	+ 0.221	(0.081)	2.73	0.007	0.062, 0.380
2016	− 0.209**	(0.100)	− 2.09	0.038	− 0.406, − 0.012
2017	− 0.069	(0.132)	− 0.52	0.601	− 0.329, 0.191
2018	+ 0.398**	(0.175)	2.27	0.024	0.053, 0.743
2019	+ 0.602	(0.185)	3.26	0.001	0.238, 0.965
2020	+ 1.061	(0.231)	4.59	0.000	0.606, 1.516
2021	+ 1.028	(0.233)	4.40	0.000	0.569, 1.488
2022	+ 1.264	(0.225)	5.61	0.000	0.820, 1.708
2023	+ 1.659	(0.275)	6.04	0.000	1.118, 2.200
Constant	7.943	(0.757)	10.49	0.000	6.451, 9.434
Observations	2225				
Groups	252				
*R*^*2*^ (within)	0.058				
*ρ* (intra-class)	0.615				

Robust SEs clustered by local authorities

The estimated fixed-effects coefficient should be interpreted as an associational parameter rather than a causal effect. Specifically, it represents the average within-local-authority association between annual changes in PM₂.₅ concentrations and suicide mortality after accounting for unobserved time-invariant local characteristics and common year-specific shocks. The estimated coefficient for PM₂.₅ is positive and statistically significant (*β* = 0.221, *p* = 0.007), indicating that a one-unit increase in annual mean PM₂.₅ concentration is associated with an increase of approximately 0.22 suicides per 100,000 population. Given a national average suicide rate of around 10 per 100,000, this corresponds to a relative increase of approximately 2–3%. Although this association remains statistically significant after controlling for local authority and year fixed effects, it may still reflect the influence of unmeasured time-varying factors that were not included in the model and should therefore be interpreted as evidence of association rather than causation.

The estimated year effects provide additional insight into temporal dynamics. Relative to the 2015 baseline, coefficients for 2016 and 2017 are negative or statistically insignificant, indicating limited change in suicide rates during this period. From 2018 onwards, however, the coefficients become positive and statistically significant, with effect sizes increasing over time. By 2019, suicide rates were approximately 0.60 per 100,000 higher than in 2015, rising to 1.66 per 100,000 by 2023. These patterns are consistent with national statistics documenting a sustained increase in suicide mortality from 2018.

Model diagnostics provide additional context for interpreting the results. The within *R*^*2*^ of 0.058 indicates that PM₂.₅ and year effects jointly explain approximately 6% of the variation in suicide rates within local authorities over time. Although this explanatory power is modest, it is consistent with expectations given the multifactorial nature of suicide. The intra-class correlation coefficient (*ρ* = 0.615) suggests that more than 60% of the overall variation in suicide mortality is attributable to persistent, time-invariant differences across local authorities, such as long-standing socioeconomic conditions and demographic structures.

Hence, Table 2 provides two principal findings. First, higher PM₂.₅ concentrations are positively and significantly associated with increased suicide rates within local authorities over time. Second, broader temporal dynamics, captured by year effects, indicate a sustained increase in suicide mortality from 2018 onwards, consistent with national trends linked to structural pressures and the COVID-19 pandemic. These results suggest that while air pollution contributes to variation in suicide risk, its effect is relatively modest compared to the influence of underlying structural conditions and wider societal shocks.

To examine potential non-linearities, Table 3 presents the results from a fixed-effects regression model that includes both linear and squared terms for PM₂.₅. The coefficient on the linear term is negative (*β* = −1.402, SE = 0.345, *p* < 0.01), while the squared term is positive (*β* = 0.090, SE = 0.019, *p* < 0.01), indicating a statistically significant U-shaped relationship between PM₂.₅ concentrations and suicide mortality. This pattern suggests that at lower levels of exposure, increases in PM₂.₅ are associated with declining suicide rates; however, beyond a threshold of approximately 7.8 µg/m^3^, further increases in pollution are associated with rising suicide mortality. The inclusion of the quadratic term leads to a modest improvement in model fit, with the within *R*^*2*^ increasing to 0.081 relative to the linear specification, indicating that accounting for non-linearity captures additional variation in suicide rates within local authorities over time*.*

**Table 3:** Nonlinear fixed-effects regression of suicide rates on PM₂.₅ (2015–2023)

	Coefficient	Std. Err	*p*-value	95% CI
PM₂.₅	− 1.402	0.345	0.000	− 2.08, − 0.72
PM₂.₅^2^	+ 0.090	0.019	0.000	0.054, 0.127
Constant	15.10	1.67	0.000	11.81, 18.38
Year effects	Included			
Observations	2225			
Groups	252			
*R*^*2*^ (within)	0.081			

Robust SEs clustered by local authorities

At lower levels of PM₂.₅, increases in pollution are associated with reductions in suicide rates. However, beyond a critical threshold, the relationship reverses, with higher pollution levels corresponding to increased suicide mortality. The estimated turning point is approximately 7.8 µg/m^3^, calculated as − β₁/(2β₂). Below this threshold, relatively cleaner local authorities tend to exhibit higher suicide rates, whereas above it, further increases in particulate exposure are consistently associated with elevated suicide risk.

The regression intercept (15.10, *p* < 0.001) has limited substantive interpretation because it represents the predicted suicide rate when PM₂.₅ equals zero, a value outside the observed range of the data. Consequently, interpretation focuses on the estimated coefficients for PM₂.₅, the quadratic term, and the calculated turning point, which provide the substantive evidence regarding the nonlinear association between particulate matter and suicide mortality. Although not reported in the table, the year fixed effects remain jointly significant and capture the upward national trend in suicide rates observed from 2018 onwards, including the pronounced increases during and following the COVID-19 period.

Model fit improves relative to the linear fixed-effects specification presented in Table 2. The within *R*^*2*^ increases from 0.058 to 0.081, indicating that allowing for a nonlinear functional form captures additional variation in suicide rates within local authorities over time. This improvement suggests that the relationship between PM₂.₅ and suicide is not constant but varies across the exposure distribution. In relatively low-pollution areas, the estimated association suggests that further reductions in PM₂.₅ are not accompanied by lower suicide rates and may coincide with higher observed rates. This pattern should not be interpreted as indicating that cleaner air increases suicide risk, but rather that other contextual factors are likely to dominate suicide mortality in these lower-exposure settings. In contrast, in more polluted urban settings, such as London boroughs, additional increases in PM₂.₅ beyond the estimated threshold of approximately 7.8 µg/m^3^ are consistently associated with higher suicide mortality, indicating a more pronounced risk at elevated exposure levels.

## Discussion

The objective of this research was to explore whether sustained exposure to PM₂.₅ correlates with higher suicide rates across UK local authorities, despite overall improvements in national air quality. The results complicate this assumption. Some model specifications reveal a positive within-area association between PM₂.₅ and suicide mortality, while others produce weak or even negative links, showing that the strength of the relationship shifts depending on model choice and time frame. Rather than indicating a direct or causal relationship, the findings suggest that the association between PM₂.₅ and suicide mortality is conditional and may partly reflect unmeasured time-varying socioeconomic influences. Although local authority and year fixed effects account for persistent regional characteristics and common national shocks, other annually varying determinants of suicide were not explicitly modeled. Consequently, the results should be interpreted as evidence of association rather than causation.

The most significant initial observation is the divergence between cross-sectional and fixed-effects results. The raw scatterplot suggested a negative correlation: more polluted areas appeared to have lower suicide rates. This pattern likely reflects structural geography. Urban boroughs such as Westminster, Camden, and Tower Hamlets consistently record higher PM₂.₅ but also benefit from dense healthcare infrastructure, better access to social services, and younger demographics, all of which are protective against suicide. By contrast, rural authorities in Wales or the Southwest, with some of the cleanest air in the country, display disproportionately higher suicide rates. These regions are marked by economic deprivation, outmigration of young adults, limited mental health provision, and higher proportions of at-risk groups such as middle-aged men. The negative bivariate association should not be interpreted as evidence that higher pollution reduces suicide mortality. Rather, it is likely to reflect the spatial clustering of pollution exposure and broader regional socioeconomic characteristics that differ systematically between urban and rural areas.

Once these persistent differences are controlled by using fixed effects, the association flips. Within a given local authority, years with higher PM₂.₅ levels tend to coincide with higher suicide rates. This suggests that while structural deprivation explains much of the cross-sectional variation, pollution may contribute to a contemporaneous physiological or psychological influence within regions. The positive coefficients are modest, equivalent to roughly a 2–3% rise in suicide deaths for each 1 µg/m^3^ increase in PM₂.₅, but meaningful when scaled to populations. This duality underscores an important implication: environmental exposures interact with place-based inequalities rather than operating in isolation. Researchers and policymakers must therefore be cautious in interpreting aggregate correlations without accounting for spatial structure.

The nonlinear model provides further nuance, revealing a U-shaped curve with a turning point at approximately 7.8 µg/m^3^. Below this threshold, cleaner areas paradoxically record higher suicide rates, while above it, additional pollution correlates with increased risk. This suggests that the effect of PM₂.₅ on suicide is not monotonic but contingent. For already-clean rural authorities, further reductions in pollution are unlikely to reduce suicide mortality, because air quality is not the binding constraint on health outcomes. Instead, socioeconomic disadvantages, social isolation, and lack of services remain the dominant drivers. In more polluted regions, however, once PM₂.₅ surpasses the 7–8 µg/m^3^ threshold, particulate exposure may meaningfully exacerbate suicide risk through biological pathways such as neuroinflammation and oxidative stress.

These exploratory findings should be interpreted with caution and require confirmation in future studies incorporating additional socioeconomic controls and alternative model specifications. Nevertheless, if confirmed, they may have implications for understanding how environmental exposures interact with broader determinants of mental health.

The nonlinear findings add further complexity to the existing literature. Previous studies conducted in South Korea have reported positive associations between particulate matter and suicide following short-term increases in pollution (Cho et al. 2014; Kim et al. 2010). Likewise, Sun et al. (2020) identified a positive spatial association between PM₂.₅ exposure and suicide mortality across London boroughs after accounting for socioeconomic deprivation (Sun et al. 2020). Our findings broadly support the existence of a positive association within local authorities while additionally suggesting that the relationship may be nonlinear, with stronger associations emerging at higher pollution levels.

In contrast, the present study finds that, at the national level, cross-sectional associations are negative, whereas within-local-authority temporal variation is positively associated with suicide mortality. This contrast implies that results drawn from highly polluted contexts such as Seoul, or from localized urban settings like central London, may not translate directly to a more varied national context.

The results, therefore, highlight the importance of spatial scale. Pollution may be a risk factor in dense urban areas with persistently high concentrations, but at the national level, geographic and socioeconomic confounding can dominate. Future research should explicitly model these interactions, perhaps by stratifying analyses between urban and rural regions or by testing for effect modification by deprivation levels.

The present analysis evaluates contemporaneous annual associations and therefore does not capture potential delayed effects of PM₂.₅ exposure. While biological mechanisms suggest that both acute and cumulative exposure pathways may exist, the available annual panel was not sufficiently long to estimate lagged relationships reliably. Future studies using longer time series or higher-frequency data should examine whether pollution exerts delayed effects on suicide mortality.

### Policy implications

The findings of this study carry important implications for public health policy, particularly in advancing integrated frameworks that link environmental exposure with mental health outcomes. Although the association between PM₂.₅ and suicide mortality is modest and context-dependent, the results indicate that air pollution should not be viewed solely as a physical health risk, but also as a potential contributor to mental health vulnerability under certain conditions.

First, the identification of a positive within-area association suggests that short-term fluctuations in air quality may influence mental health outcomes. While the magnitude of this effect is smaller than that of broader socioeconomic drivers, it remains meaningful at the population level. This supports incorporating mental health co-benefits into air quality regulation, where measures such as emission controls and transport policies may contribute to both physical and psychological health improvements.

Second, the observed nonlinear relationship highlights the relevance of threshold-based policy approaches. Mental health risks may become more pronounced beyond approximately 7–8 µg/m^3^, indicating that further pollution reductions in already clean regions may have limited impact, whereas stricter standards in higher-exposure urban areas could yield greater benefits. This underscores the value of geographically targeted interventions over uniform national strategies.

Third, the findings emphasize the importance of environmental justice. Areas with higher pollution exposure often coincide with socioeconomic disadvantages, amplifying vulnerability. Policy responses should therefore adopt place-based strategies that integrate environmental regulation with expanded mental health services and broader social support systems.

Finally, the results highlight that environmental factors operate alongside, rather than replace, broader structural determinants of suicide. Effective policy responses require a multisectoral approach that combines environmental, social, and healthcare interventions to address the complex drivers of mental health outcomes.

## Conclusion, limitations, and future research

This study provides national-level evidence on the relationship between fine particulate matter (PM₂.₅) and suicide mortality across UK local authorities between 2015 and 2023. The findings do not support a simple or uniform association. While bivariate analysis suggests a negative relationship, fixed-effects models reveal a modest but statistically significant positive association within local authorities. Nonlinear specifications further demonstrate a U-shaped relationship, with suicide risk increasing beyond an estimated threshold of approximately 7.8 µg/m^3^. Overall, the results suggest that PM₂.₅ is associated with suicide mortality in a conditional and context-dependent manner, with the observed associations varying across exposure levels and geographic settings. Given the observational design and the absence of certain time-varying socioeconomic controls, these findings should be interpreted as exploratory associations rather than evidence of causality.

Several limitations should be acknowledged. First, the analysis does not incorporate local authority-specific time-varying socioeconomic variables such as unemployment, housing stress, mental health service provision, or substance use because consistent annual data covering all authorities were unavailable. Consequently, residual confounding cannot be excluded, and the reported estimates should be interpreted as associative rather than causal. Second, the use of annual average PM₂.₅ concentrations may mask shorter-term exposure effects relevant to acute behavioral outcomes. Third, the ecological study design precludes inference at the individual level and does not account for personal risk factors such as psychiatric history or socioeconomic status. Finally, the relatively modest within *R*^*2*^ values reinforce that suicide is influenced by numerous interacting determinants beyond air pollution.

Future research should incorporate higher temporal resolution data (e.g., monthly or daily observations), longer longitudinal time series, and lagged or distributed-lag modelling approaches to examine whether PM₂.₅ exposure influences suicide mortality over delayed time horizons.

## Data Availability

All data sources are disclosed in this research.
